# Indexing climatic and environmental exposure of refugee camps with a case study in East Africa

**DOI:** 10.1038/s41598-023-31140-7

**Published:** 2023-05-09

**Authors:** Michael Owen, Andrew Kruczkiewicz, Jamon Van Den Hoek

**Affiliations:** 1 Department of Earth and Environmental Sciences, Columbia University, New York, 10027 USA; 2 International Research Institute for Climate and Society, Climate School, Columbia University, New York, 10027 USA; 3 Red Cross Red Crescent Climate Centre, The Hague, The Netherlands; 4 Faculty of Geo-information Science and Earth Observation, University of Twente, Enschede, The Netherlands; 5 Geography and Geospatial Science Program, College of Earth, Ocean, and Atmospheric Sciences, Oregon State University, Corvallis, 97331 USA

**Keywords:** Climate-change impacts, Natural hazards, Environmental impact, Climate-change impacts, Environmental impact

## Abstract

This study presents a novel approach to systematically measure climatic and environmental exposure in refugee camps using remote sensing and geospatial data. Using a case study of seventeen refugee camps across five countries in East Africa, we develop a climatic and environmental exposure index to quantify each camp’s exposure relative to a population of simulated camp locations within the hosting country. Our results show that seven of seventeen refugee camps are within the upper two quartiles of exposure relative to a simulated population, suggesting that more than six-hundred thousand refugees living in these camps face elevated exposure compared to other potential camp locations. This method stands to improve the process of gathering and analyzing climatic and environmental data on geographically remote humanitarian spaces in a reliable, low-cost, and standardized manner. Automation and refinement of this index could enable real-time updates on climatic and environmental exposure to support decision-making related to disaster risk reduction in refugee camp management.

## Introduction

Climate change research has long aimed to quantify the vulnerability of human populations to natural hazards by considering the intersection of a population's social characteristics and environmental context^1^. Seminal literature has modeled natural hazard vulnerability quantitatively and qualitatively based on various factors (e.g., environmental quality, economic activity, health/sanitation outcomes, educational attainment, demographics), but such assessments are often carried out at a regional or sub-national scale without consideration of a population’s socioeconomic or geographic marginalization, or localized climatic and environmental extremes such as heat waves or precipitation variability^2,3^. As a result, such assessments may misrepresent the magnitude, timing, and manner of a population's vulnerability, thus concealing the effects of climate change^4^ and potentially lead to maladaptive approaches to mitigate actual population- or place-specific vulnerability^5,6^. Even when vulnerability modeling is localized, the variation in geospatial inputs or assumptions across models may challenge comparison over time or between populations, thereby impeding systematic awareness of vulnerabilities^7^. Vulnerability assessment has advanced in recent years, particularly at local and community levels as increased availability of high spatial and temporal resolution in situ data becomes easier to collect and access^8–10^. Further, regional and global scale approaches have been developed, such as the MultiRISK Modeling and the Visualization Tool^11^; however, limitations exist particularly in consistency in data quality across a given study area (such as with landslides^11,12^).

The need for tailored, systematic vulnerability assessments is arguably the most acute for refugee camp settings, which are broadly characterized by data scarcity or outright absence^13,14^. In planning refugee camps or evaluating potential hazards, refugee camp managers and humanitarian organizations make prioritization and logistic decisions on local to regional scales, often with a lack of high quality, high spatial resolution data. As of mid-2022, there are approximately 26 million refugees under UNHCR mandate across 120 countries, with the majority of refugees living in regions that have witnessed relatively large changes in temperature or precipitation regimes across the Middle East, Sub-Saharan Africa, and South Asia^15^. Refugees are commonly settled in environmentally marginal borderlands^16^, excluded from socioeconomic opportunities and livelihoods^17^, and may remain encamped for generations^18^. While refugees are one of the most studied at-risk populations from a social vulnerability perspective^18–20^, refugee camps have only been comprehensively studied in terms of climate vulnerability and environmental change studies in a few cases. For example, the rapid and widespread landscape changes (e.g., deforestation, terracing) and related hazards (e.g., landslides, flooding) in Cox's Bazar, Bangladesh, home to the world's largest refugee population of one million Rohingya has received extensive attention^21–23^. In other refugee settings, land cover change assessments have found a greater amount of land degradation on the periphery of and within refugee camps in Sudan^24^, South Sudan^25^, Uganda^26^, and broader East Africa^27^. Maystadt et al.^28^ found a pattern of agricultural deforestation in refugee-hosting regions across continental Africa, while Solemi^29^ found that encamped refugee populations do not disproportionately contribute to regional deforestation. Considering climatic conditions, Van Den Hoek^30^ modeled future climate projections for temperature and precipitation change under RCP 8.5 in the year 2041–2060 and found that current refugee camps are projected to see comparable changes in surface temperature and much higher changes in precipitation compared to nearby reference locations. By comparison, there are relatively few refugee camp-level vulnerability studies that analyze the risk of climate- or weather-related hazards to refugee populations^31^ (with exceptions^22,32,33^).

While the need to minimize the exposure of camps to extreme climate conditions and mitigate climate and environmental risks^34^ is recognized, climate risks are only briefly mentioned in UNHCR’s Camp Site Planning Minimum Standards documentation^35^, and minimum standards for climatic and environmental exposure assessments have yet to be adopted by UNHCR. UNHCR and refugee hosting countries make limited use of environmental assessments in planning refugee camps^6^, gauging potential impacts of climate change^36^ or the frequency of natural hazards in refugee camps^37^. At the camp-level, refugee camp managers rarely incorporate structured approaches to climate-informed decision-making^6,38^, this is not necessarily due to resource constraints or a lack of expertise but instead often results from an oversaturation of climate and geophysical products^39^ and an absence of a framework that integrates relevant environmental and climatic datasets to inform spatially explicit and timely decisions^40^.

As global refugee populations and the impacts of climate change continue to rise, there is a critical need to make better use of available environmental and climate data to mitigate future risk for encamped refugee populations^15^. In this paper, we present a novel climate and environmental exposure index for refugee camps in East Africa that considers primary, regional natural hazards including floods (both riverine and intense rainfall induced), drought, extreme heat, and landslides (including mudflows)^41^. This index is based on eleven satellite remote sensing-based products and tailored to support refugee camp planning, siting, and management decisions, including those related to disaster risk reduction and anticipatory action. The index calculation is informed by exposure and vulnerability mapping methodologies^42^, climate migration modeling approaches^43^, and considerations in refugee camp siting and management^44^, and it is intended to be extended to a variety of geographic contexts. We evaluate the index at seventeen refugee camps in five countries in East Africa, and compare exposure to a sample of sites in surrounding national border regions, which commonly host refugee camps. Next, we present the exposure index calculation, quantify the exposure of refugee camps relative to sampled non-refugee locations, rank and identify the most prominently exposed refugee camps relative to regional conditions, and offer policy recommendations for improved uptake of climate and environmental datasets in refugee camp-specific planning and management.

## Results

We found that most study refugee camps are clustered around the median exposure of sample border sites within their respective country (Table 1). Kakuma refugee camp (Kenya) is the only camp near the top quartile (70%) of national exposure and is also home to the fourth largest encamped refugee population in the world. Six other refugee camps are in the second highest quartile of national exposure, with Pamir (South Sudan) having the highest exposure at 67%, and five other camps clustered from 57 to 51% (Ajuong Thok, Yida, Ifo, Hagadera, and Nyarugusu). These seven camps in the third quartile are home to approximately 677,820 (41%) of the total population across study refugee camps. Seven refugee camps are in the second lowest quartile of exposure, comprising 735,165 refugees (44% of the study population). The remaining three refugee camps occupy the lowest exposure quartile, comprising 228,565 refugees (13% of the study population). For refugee camps across the five study countries, South Sudan and Kenya had the highest relative exposure (both average and median), while Tanzania and Uganda had the lowest relative exposure.

**Table 1:** Ranked refugee camp exposure and main drivers.

Refugee camp	Country	Population (2020)	Year established	Percentile of national exposure (CI)	Key drivers of exposure
Kakuma	Kenya	185,000	1992	69.8 (62.6–72.1)	TXx, PDSI, SSM
Pamir	South Sudan	27,489	2018	67.5 (63.1–71.7)	PDSI, SSM
Ajuong Thok	South Sudan	55,000	2013	57.7 (52.5–61.7)	PDSI, SSM, TXx
Yida	South Sudan	70,331	2012	55.3 (49.5–59.1)	PDSI, TXx, SSM
Ifo	Kenya	84,000	1992	52.8 (44.9–56.8)	SSM, TXx
Hagadera	Kenya	106,000	1992	51.4 (42.9–55.6)	TXx
Nyarugusu	Tanzania	150,000	1996	50.9 (44.3–53.3)	ΔT, TXx
Melkadida	Ethiopia	34,762	2010	44.7 (37.7–49.7)	Q, TXx, SSM
Dagahaley	Kenya	87,000	1992	36.6 (28.5–40.9)	SSM, TXx
Pugnido	Ethiopia	62,000	1993	35.5 (31.3–39.9)	Q
Palorinya	Uganda	166,025	2016	34.7 (29.7–39.1)	Q, SSM
Nyumanzi	Uganda	52,894	2014	32.5 (28.3–36.9)	Q
Bidi Bidi	Uganda	287,087	2016	28.5 (24.4–32.5)	Q, TXx
Kule	Ethiopia	45,397	2014	25.9 (21.6–29.5)	Q, TXx
Nguenyyiel	Ethiopia	83,658	2016	24.4 (20.2–27.9)	Q
Nduta	Tanzania	104,784	2015	23.6 (19.2–27.7)	ΔT, P_MAX_
Mtendeli	Tanzania	40,123	2016	15.2 (12.0–18.6)	ΔT

The Percentile of National Exposure is the measured exposure for each camp, the confidence interval is derived from the empirical bootstrap sample in Fig. 8

PDSI, Palmer drought severity index; P_MAX_, Precipitation maximum monthly anomaly in MAM (March–April–May) season; Q, Specific humidity; SSM, Surface soil moisture (driven by low SSM); TXx, Annual daytime maximum surface temperature; ΔT, Long-term temperature anomaly.

**Figure 1:**
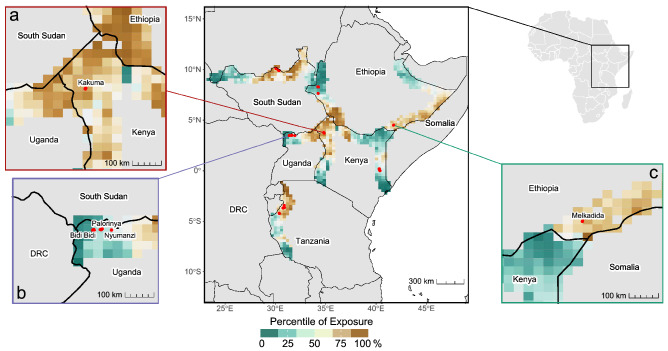
Geographic distribution of exposure across border regions. Exposure is measured as the mean exposure of quarter-degree grid cells (approx. 28 km^2^ at the equator) at refugee camps or sampled border sites. Inset Map A includes Kakuma refugee camp in Kenya, and spans northeast Uganda, northern Kenya, and southwest Ethiopia. Inset Map B includes Bidi Bidi, Nyumanzi, and Palorinya refugee camps in northwest Uganda, and spans eastern Democratic Republic of Congo, southern South Sudan, and northwest Uganda. Inset Map C includes Melkadida refugee camp in Ethiopia, and spans northeast Kenya, southeast Ethiopia, and southern Somalia. Map created in R Studio 4.2.2.

We also identified patterns of exposure in national border regions that have a bearing on refugee camps and sampled border sites. As each exposure index is normalized at the country level, in-country and in-region exposure hotspots can be identified by using the sample data as the basis for binned heatmaps. For example, the border region (Fig. 1a) between northeast Uganda, northern Kenya, and southwest Ethiopia has the highest mean exposure of 0.46 (standard deviation: 0.04; median percentile: 75th relative to each country) of any border region in the study area. The heightened overall exposure is further evidenced by the country-level percentile for each sample site (n = 1305) within this border region. The country-sample median (Fig. 1a) ranges from the 73rd to 88th percentile (country-level mean exposure range from 0.45 in Kenya to 0.48 in Ethiopia), indicating that this region between Uganda, Kenya, and Ethiopia is the most exposed border region for each of the three countries.

Beyond the refugee camps that are enveloped in a region of comparable exposure, such as Kakuma (Kenya); others are located in transitional regions, such as the three moderately exposed refugee camps Bidi Bidi, Nyumanzi, and Palorinya (Uganda) 200 km west of the high exposure region that includes Kakuma (Fig. 1b). Similarly, the Melkadida refugee camp (Ethiopia) (Fig. 1c) is located in a moderately high-exposure region just 50 km east of a pocket of relatively low exposure in Kenya.

We also identified the key variables that drive exposure as variables that satisfy the two following conditions: (1) have one of the three highest normalized values at a given camp, and (2) fall within the top quartile of the national distribution of values at sampled border sites. Considering the variation in key drivers across study camps, we show that the exposure of a given refugee camp is representative of localized environmental and climatic conditions, rather than simply the exposure in the surrounding border region. For example, the percentile of national exposure at two of the three camps that make up the Dadaab refugee complex in Kenya—Ifo and Hagadera—was at least 10 percentile points higher than the exposure at the third Dadaab camp, Dagahaley, despite being located only 4.4 km away; the key drivers of exposure (SSM and TXx) were shared between Ifo and Dagahaley, with TXx as the sole key driver for Hagadera.

We found three key drivers of exposure in approximately half of the study camps: annual daytime maximum surface temperature (TXx; 10 of 17 camps), low surface soil moisture (SSM; 8 of 17 camps), and specific humidity (Q; 7 of 17 camps). The Palmer Drought Severity Index (PDSI), the long-term temperature anomaly (ΔT), and the precipitation maximum monthly anomaly in the March–April-May season (P_MAX_) were only identified as key drivers of exposure in four, three, and one camp(s), respectively. Still, these camps include Kakuma and Pamir, which had the two highest overall exposures. Other than SSM, none of the geophysical variables were identified as being key drivers of exposure, indicating little difference in geophysical conditions between refugee camps and sample border locations. Interestingly, the three most common key drivers also have the largest spatial resolutions of the eleven variables (ranging 0.1 to 2.5 deg), showing that meaningful differences between climate, weather, and geophysical conditions at refugee camps and sampled border sites are evident even with coarser resolution variables.

**Figure 2:**
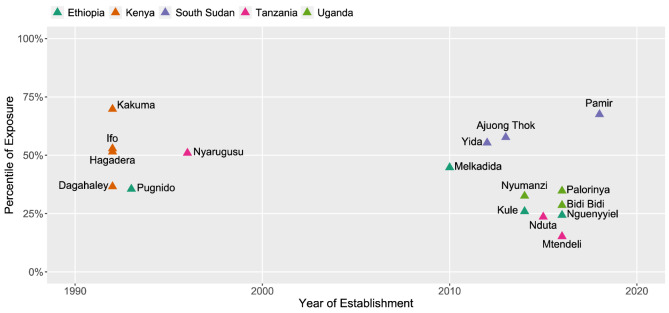
Percentile of exposure and year of refugee camp establishment.

Although there is little correlation between the percentile of exposure and the year of camp establishment (R^2^ = 0.16), there is a marked difference in exposure between camps established pre-2000 (n = 6, mean percentile of exposure: 49.5) and post-2000 (n = 11, mean percentile of exposure: 37.3) (Fig. 2). Kakuma refugee camp stands out as having the greatest exposure of all camps in the study in the 70th percentile. Kakuma is also the oldest camp, formally established in 1992. While identifying potential reasons for differences in camp exposure over time are outside the scope of this study, it is worth noting this distinction given the evolution of camp planning by UNHCR and other actors in the humanitarian space^34,45^ in recent decades. Shifts in the geography of conflict and unrest over time have also driven displacements into new border regions, with varying responses from host communities/governments pushing refugee camps into further marginal borderlands^20^. The data also indicates a rise in the variability in exposure of camps settled since 2010 with many of the lowest and highest exposure camps having been settled in the last decade.

**Figure 3:**
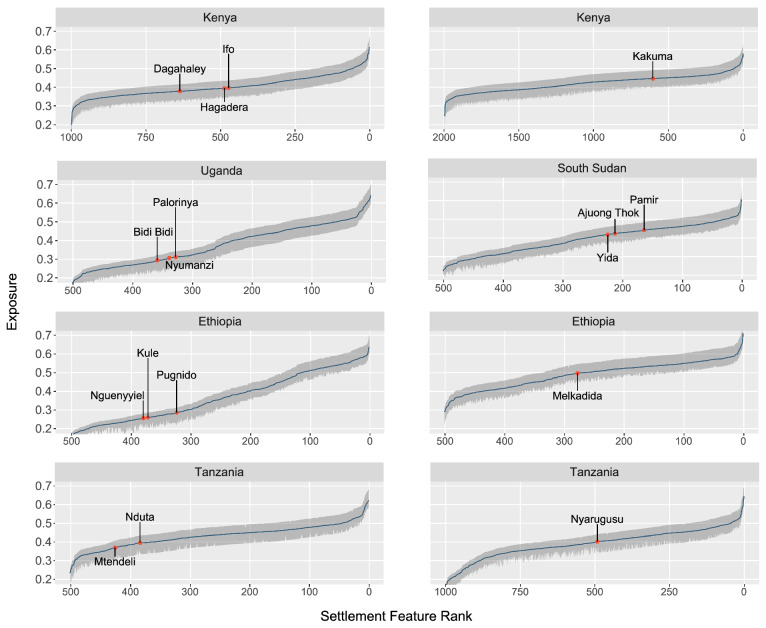
Exposure distributions by camp and reference sample. The sensitivity ribbons (in gray) represent the upper and lower bounds of exposure across eleven exposure variants.

The sensitivity analysis of iteratively removing variables to gauge the influence of a given variable on the overall exposure showed that the exposure rank of each camp was only marginally sensitive to the exclusion of any variable (Fig. 3). Across all countries, the maximum sensitivity across all exposure variants was 0.08 for the lower bound exposure in Kenya (shared borders: Somalia and Ethiopia) and 0.064 for the upper bound of exposure in Ethiopia and Uganda (shared border: South Sudan). The average sensitivity of the lower bound of exposure for each country was also marginal, deviating at most by 0.053 in Tanzania (shared border: Burundi) and just 0.042 in Ethiopia (shared border: Somalia); the sensitivity of the upper bound was also consistent and low, deviating at most by 0.048 in Ethiopia (shared border: Somalia) and just 0.037 in Ethiopia and Uganda (shared border: South Sudan). Within the study camps, the most significant variable in terms of potential deviation in exposure was the Annual Daytime Maximum Surface Temperature (TXx).

In addition to testing the sensitivity of individual variables, we also generated an empirical bootstrap sample based on 10,000 model iterations to estimate confidence intervals per camp (Fig. 8). The average 95% confidence interval of the percentile of exposure deviated ± 4.73% for each camp from the estimated median, at most for the three camps in the Dadaab refugee complex by ± 6.19% (Hagadera, Dagahaley, Ifo), and the least for Mtendeli (± 3.29%) and Nguenyyiel (± 3.89%).

## Discussion

To our knowledge, this research provides the first systematic multi-hazard assessment of environmental and climatic exposure across refugee camps spanning multiple countries. We found that seven refugee camps—Kakuma, Melkadida, Pamir, Ifo, Hagadera, Yida, and Ajuong Thok—across three countries (Kenya, Ethiopia, South Sudan) are more exposed to climate and environmental conditions than their respective average border sites. As climate change continues to accelerate, high confidence increases in surface temperature and pluvial flooding, among other drivers of exposure for the region of central and eastern Africa, could augment the relative exposure of refugee camps or redistribute the relative exposure across camps and surrounding border regions^46^. The index construction approach presented here can track future changes in exposure at existing camps or be adapted to guide the selection of a minimally exposed location for construction of a new refugee camp. In addition, the index can be tailored to assess exposure for all refugee camps, informing context-specific decision-making based on camp results. For example, for cases where a refugee camp is sited in an area of heightened exposure, the index calculation can be used to understand the degree to which they are exposed, the difference in exposure compared to nearby areas, and, for both of these metrics, support the monitoring of refugee camp exposure change over time. Longer-term monitoring can inform prioritizing or deprioritizing operational and risk reduction actions, even in the absence of standard operating procedures to assess the exposure of refugee camps.

Given the paucity of climate hazard data at refugee camps, a rigorous assessment of the relationship between camp-level exposure as estimated in this work and hazard incidence is not possible. While an exhaustive record of hazard events is unavailable, there has been ad hoc documentation of climate hazard events by humanitarian organizations, including UNHCR, that highlight the occurrence of drought and flood events that have been recorded over the last fifteen years in Kenya’s Kakuma refugee camp and Dadaab refugee complex (comprised of Dagahaley, Hagadera, and Ifo refugee camps) (Table 2). These documented droughts and floods likely do not represent the totality of hazard event occurrence nor the diversity of hazard types at refugee camps. Natural hazard-driven disasters and disruptions with minimal impact on refugee camp populations or infrastructure may not be as readily documented. Individual-level physiological climate hazards (i.e., heat stress) may be less documented than meteorological hazards. Nonetheless, these known hazard events are indicative of the key drivers of exposure, SSM and TXx, identified at Kakuma and the three camps that comprise Dadaab.

**Table 2:** Documented hazard events and impacts in Kenyan refugee camps.

Camp/complex	Country	Hazard event	Affected population	Year
Kakuma	Kenya	Drought	< 20 children	2022
Kakuma	Kenya	Flood	1000 individuals	2021
Kakuma	Kenya	Flood	< 350 individuals	2020
Dadaab*	Kenya	Flood	11,000 + families	2017
Kakuma	Kenya	Flood	50,000 individuals	2014
Dadaab*	Kenya	Drought	3–6 × increased infant mortality rate	2011
Dadaab*	Kenya	Floods	12,000 + individuals	2006
Kakuma	Kenya	Floods	2000 + individuals	2006

*Dadaab refugee complex includes Dagahaley, Hagedera, and Ifo refugee camps.

This study's framework supports a retrospective analysis of climate exposure in refugee camps and risk-informed or forecast-based approaches, such as anticipatory action (AA)—the act of prioritizing when and where to take specific actions based on uncertain forecast information^47^. The work here can directly inform when and where actions should be taken—and perhaps more importantly, when and where actions must be deprioritized due to limited resources. For existing refugee camps, baseline and periodic assessments of climate exposure can inform the prioritization of refugee camps for AA implementation. For example, after a climatic shock, such as a flash flood, occurs in a refugee camp, determining the degree to which weather, climate, and physiographic factors contributed to the shock would be valuable information in prioritizing when and where recovery and resilience actions should take place, and perhaps to consider which areas should be abandoned due to heightened risk. Assessments of baseline risks like those presented here are also valuable in evaluating potential sites for a future refugee camp to understand the extent to which climate shocks and hazards could make refugees vulnerable if action is not taken.

This study has several limitations that are worth mentioning. The reliance on long-term temporal averages of satellite-derived products with coarse spatial resolutions inevitably overlooks short-term variations in exposure and long-term exposure trends. Reliance on these derived products also limits the ability to detect spatial variability in exposure within a given refugee camp, given the resolution. The sensitivity of index outcomes to the spatial resolution of aggregation and temporal scales could be further investigated, although initial testing found marginal effects on overall camp exposures (Fig. 3). As mentioned above, the general unavailability of climate hazard event documentation across study refugee camps prevents empirical validation of the relationship between modeled exposure and hazard events. Having data on camp management decision-making during periods of heightened risk of a natural hazard and the coping strategies of refugee populations would also help situate the theoretical framing of the exposure index. The selection of specific variables is context-dependent and may need to be adapted if the framework were to be deployed in another geography with different potential drivers of exposure or hazards. Similarly, since the area of sample border sites (3.1 km^2^) is more representative of the typical area of refugee camps in East Africa rather than the area of the seventeen study camps, which are among the largest in East Africa (mean boundary area: 7 km^2^), the area of the region surrounding the non-refugee sites selected for comparison may need to be adjusted, or the sensitivity of exposure measurements to the selected radial buffer width could be assessed.

Future applications of this framework would benefit from forming a more rigorous connection between camp-level refugee demographics, infrastructure, documentation of adverse weather events/hazards and their impacts, and the causal pathways for impacts on encamped refugee populations. Bringing these data together would not only help to calibrate or validate the exposure index in a specific geographic context, but it would also aid in the understanding of how refugee camp characteristics and management decision-making can mitigate exposure and prevent hazards from becoming disasters—including early warning and prioritization of anticipatory actions based on forecasts.

Given that the global refugee population has steadily grown in recent years and a typical refugee can expect to stay in a camp for a decade or more, camp management strategies, especially in protracted refugee situations, must be more responsive to current and future exposure^48,49^. This research offers three contributions to climate-sensitive decision-making in refugee contexts: a quantitative, integrated assessment of the environmental and climatic exposure of refugee camps relative to other camps and border sites within each country; identification of the main drivers of exposure in each refugee camp and across a country; and methods that are designed to be iterated in novel refugee contexts with potentially different drivers of exposure, which has been a key limitation of previous studies on refugee camp exposure^50^. While the location and management of refugee camps is driven by a variety of geophysical, climatic, and socioeconomic factors, including logistic and political concerns associated with aid distribution and land tenure^51,52^, this research offers a new way to assess the exposure implications of camp site selection and hopefully will inform more climate-sensitive camp planning and management practices going forward.

## Materials and methods

### Climatic and environmental exposure index conceptualization and variable selection

As a first step, we create an index of climatic and environmental exposure that integrates spatially referenced data representing climate, weather, and geophysical conditions. The conditions that are prioritized in this study are defined by the hazards of interest for the region, including temperature-related and hydrometeorological. With floods and landslides usually driven at least in part by above-average precipitation, it was important to include precipitation as a variable of interest. For flash floods in particular, although sub-daily rainfall data would be ideal, rainfall at this time step in the region varies considerably spatially and seasonally^53^, therefore a coarser approach to rainfall variability is taken, using seasonal anomalies, to allow for regional analysis.

**Figure 4:**
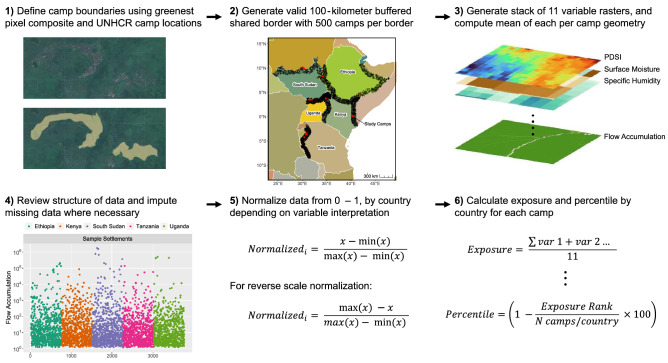
Six-step framework for creating exposure index and percentile at refugee camps. Map created in R Studio 4.2.2.

For a given camp, we measure the exposure index, compare the camp’s exposure value to the distribution of exposure values measured at a stratified sample of locations in the border region of the camp’s country—where refugee camps are commonly established—following Van Den Hoek^30^ and de Sherbinin et al.^42^, and identify the camp’s percentile of exposure (Fig. 4).

The variables for the exposure index (Table 3) were sampled from a more extensive list of exposure variables (e.g., land cover change, temperature and precipitation maxima, and fire and water occurrence) from vulnerability mapping scholarship^42^ and well-documented exposure risks in the refugee-hosting region of Cox’s Bazar in Bangladesh^6^. Selected climate variables capture historical extremes and patterns, which embed the current and future exposure of a refugee camp if trends continue. Weather variables were chosen to capture the central tendencies of precipitation and temperature, which, when elevated, can drive exposure for refugee populations^54^. Geophysical variables capture topographic and physiographic conditions at and surrounding camp locations, which have a bearing on natural hazards^55^. Only variables observed within study refugee camps were considered in the index calculation, while variables that have not historically affected study refugee camps were excluded. Finally, if a variable was found to be highly correlated (R > 0.75) with another variable but less representative of the specific hazard, it was also removed from the index calculation (e.g., subsurface soil moisture was highly correlated with surface soil moisture, but only surface soil moisture was included)^56^.

**Table 3:** Exposure index variables and associated information.

Variable	Phenomenon	Units	Observation period	Spatial resolution	Source
Climate and weather					
PDSI average	Drought	(unitless)	2000–2020	0.04°	TerraClimate Palmer Drought Severity Index
Precipitation: change in annual accumulation	Drought/Flood	mm/pentad	1981–2020	0.05°	UCSB CHIRPS Pentad Annual Average Precipitation change
Precipitation: interannual coefficient of variation	Drought/flood	mm/pentad	1990–2020	0.05°	UCSB CHIRPS
Precipitation: maximum monthly anomaly in MAM (March–April–May) season (P_MAX_)	Flood	mm/pentad	2000–2020	0.05°	UCSB CHIRPS
Temperature: annual daytime maximum surface temperature (TXx)	Drought	Kelvin	2010–2020	2.5°	CFSV2: NCEP Climate Forecast System Version 2, 6-Hourly Products; Maximum temperature 2 m above ground, 6-h interval (12:00)
Temperature: long-term anomaly (ΔT),	Drought	Kelvin	1951–2020	2.5°	NCEP/NCAR Reanalysis Data, Surface Temperature
Specific humidity (Q)	Drought	kg kg-1	2000–2020	0.1°	FLDAS: Famine Early Warning Systems Network
Geophysical					
Flow accumulation	Flood	(derived)	N/A	0.004°	WWF HydroSHEDS Flow Accumulation
Friction	Landslide	km/hr	N/A	0.01°	Oxford Global Friction Surface 2019
Slope	Landslide	meters	N/A	0.001°	SRTM Digital Elevation Data Version 4
Surface soil moisture (SSM)	Drought	mm	2015–2020	0.1°	ASA-USDA Enhanced SMAP Global Soil Moisture Data Surface

At the equator, 0.01° is approximately equivalent to 1.11 km. The start period begins on January 1 of a given year and ends on December 31, unless otherwise stated. Variable-camp-level data is available in Supplementary Fig. S2.

The eleven selected variables have coverage from 2015 to 2020, and most have coverage since 2000, a period of considerable changes in precipitation and temperature regimes. From 1985 to 2018, the Eastern African Long Rains (March to May) shifted in duration and volume with a drier second period from 1986 to 2007 and higher variability from 2009 to 2018^57^. Mean temperatures in East Africa have risen by 0.7 to 1 °C from 1973 to 2013, with substantial diversity between countries and seasons^58^. Temperature changes have been greatest in the northern and central regions, with more significant increases in mean minimum temperatures at night. When coupled with rising daytime temperatures, such nighttime temperature increases heighten mortality and morbidity risks for vulnerable populations^59^. All variables are included in the Google Earth Engine data catalog in raster format. The datasets vary in spatial resolution from 0.001° for slope to 2.5° for the NCEP/NCAR surface temperature reanalysis data, with a median resolution of 0.05°, or 5 km at the equator.

All climate and weather variables in the index represent many years of data, which was reduced to the temporal average or coefficient of variation over the duration of the observation period. We also calculated the long-term temperature anomaly and change in annual precipitation accumulation following Eqs. (1–2). We use the standard 30-year averaging period to define a climate normal for temperature change and a shorter 20-year period for precipitation change due to data availability as CHIRPS’ data series began in 1981. The World Meteorological Organization has found that this is an acceptable period for calculating non-extreme parameters, with 10–12-year periods generating a similar predictive skill to a 30-year period^60^.

1 $$Temperature\;Anomaly = \overline{x}\left( {Temperature_{2000 - 2020} } \right) - \overline{x}\left( {Temperature_{1951 - 1990} } \right)$$

2 $$Change\;in\;Precipitation\;Accumulation = \overline{x}\left( {Precipitation_{2009 - 2020} } \right) - \overline{x}\left( {Precipitation_{1981 - 1990} } \right)$$

### Refugee camp selection

**Figure 5:**
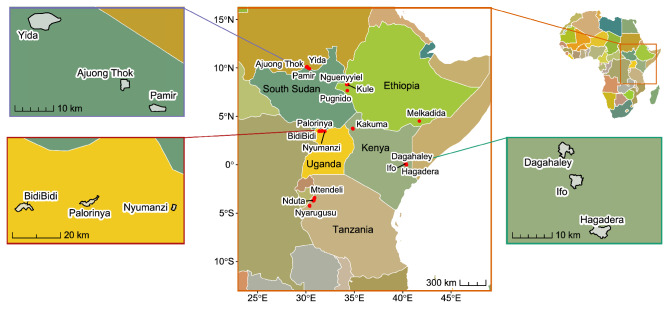
Study refugee camp locations (red dots) across five countries in east Africa. Map created in R Studio 4.2.2.

**Figure 6:**
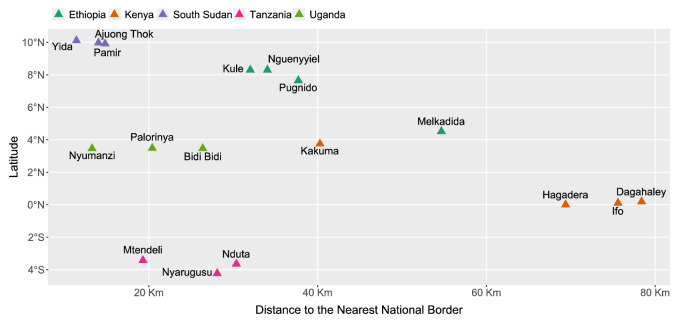
Latitude and border proximity of study refugee camps.

We considered three factors in selecting refugee camps for this analysis: climatic and environmental data availability, camp-level population data availability, and high population density^61^. With these criteria, we selected 17 refugee camps in five East African countries (Ethiopia, Kenya, South Sudan, Tanzania, and Uganda) that collectively encamp 1,641,550 people as of 2020 and include the world's second-largest refugee camp by population, Bidi Bidi (Uganda). Study refugee camps were settled between 1992 and 2016 (median: 2013), encompass a wide range of ethnic groups, nationalities, and relationships with their host communities (Table 4, Fig. 5), and are predominantly close to national borders (mean distance: 35 km) (Fig. 6). Several study camps are sited near each other, with Kenya’s Hagadera, Dagahaley, and Ifo refugee camp centroids within 8.2–22.3 km, comprising the broader Dadaab refugee complex; and Tanzania’s Mtendeli and Nduta within 21 km of each other, as well as nearby Nyarugusu refugee camp (< 75 km from Mtendeli and Nduta), each established to receive asylees from nearby Burundi and the Democratic Republic of Congo. Refugee camp centroid locations were accessed using the UNHCR Geoservices Map Portal^44^.

**Table 4:** Study refugee camp overview.

Camp	Country	Population	Established
Kule	Ethiopia	45,397	2014
Melkadida	Ethiopia	34,762	2010
Nguenyyiel	Ethiopia	83,658	2016
Pugnido	Ethiopia	62,000	1993
Dagahaley*	Kenya	87,000	1992
Hagadera*	Kenya	106,000	1992
Ifo*	Kenya	84,000	1992
Kakuma	Kenya	185,000	1992
Ajuong Thok	South Sudan	55,000	2013
Pamir	South Sudan	27,489	2018
Yida	South Sudan	70,331	2012
Mtendeli	Tanzania	40,123	2016
Nduta	Tanzania	104,784	2015
Nyarugusu	Tanzania	150,000	1996
Bidi Bidi	Uganda	287,087	2016
Nyumanzi	Uganda	52,894	2014
Palorinya	Uganda	166,025	2016
Total		1,641,550	

*Part of Dadaab refugee complex.

### Refugee camp boundary mapping

To estimate the spatial extent of land inhabited by and accessible to refugees within a given camp, we used a combination of the UNHCR-provided refugee camp centroid coordinates and a generated greenest pixel composite using Sentinel-2 NDVI (normalized difference vegetation index) satellite data from 2018 at each refugee camp. This approach is necessary as UNHCR provides camp centroid coordinates, without spatially referenced boundary data available for all study refugee camps. We manually digitized refugee camp boundaries based on the contrast between the relatively low-NDVI land within refugee camps (resulting from structures and other non-vegetated surfaces) and the surrounding land with higher NDVI. Existing UNHCR planning maps and documentation were also used to guide camp boundary interpretation. We also referenced available high-resolution satellite imagery and OpenStreetMap structure data to ensure that our interpreted boundaries encompassed all camp structures. Because several of the countries within our study restrict refugee movement beyond camp boundaries (with the notable exception of Uganda), the derived camp boundaries (mean boundary area: 7 km^2^) offer a reasonable, albeit conservative, estimate of the land directly accessible by refugees.

### Sampling border sites to compare with refugee camp exposure

To compare the exposure at study refugee camps to non-refugee camp locations in border regions of each country, we first built a dataset of valid country borders based on camp- and district-level data provided by UNHCR’s Global Public API. For each study camp, we query the most disaggregated data on the national origin of the refugee population; when camp level data is not available, we use district-level data. Each origin country that shares a border with the study camp’s country and comprises more than one percent of the total camp population presents a valid border for comparison. Given that refugee camps are not randomly distributed within or along the borders of host countries but, rather, often proximate to the origin population country’s border, this geographic subset provides an appropriate comparison for other potential camp locations (see Supplementary Fig. S1).

**Figure 7:**
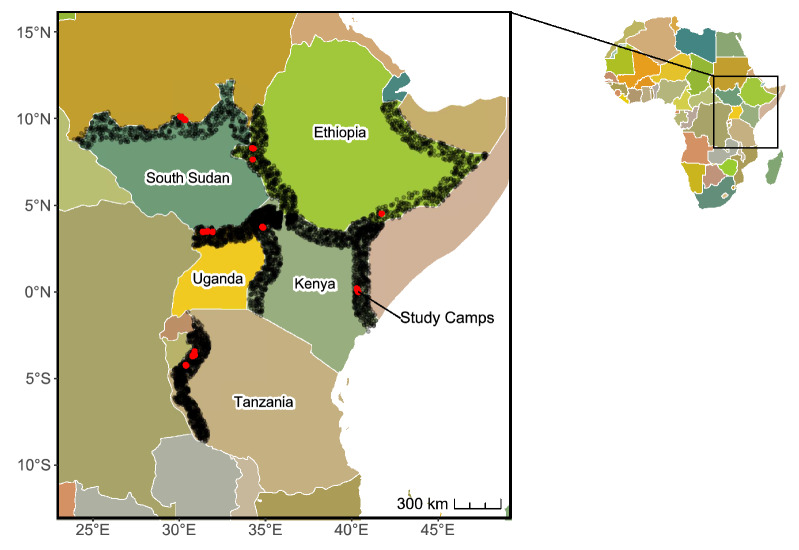
Location of sampled border sites (open black circles) for comparison with study refugee camps. Each border for comparison contains 500 randomly distributed sampled border sites (e.g., the shared border between Tanzania and Burundi contains 500 sampled border sites). Map created in R Studio 4.2.2.

We then created a 100-km-wide buffer along each valid border within each host country to approximate the near-border region that encompasses our study refugee camps (Fig. 7). We removed locations with at least one month of seasonal water based on the JRC Global Surface Water dataset^62^ as seasonally or permanently inundated sites have distinct hazard profiles compared to study refugee camps. Next, we randomly sampled 500 points within each 100-km shared border region (Fig. 7) and buffered sample locations by one kilometer to simulate the boundary of the refugee camps. Each study camp’s reference sample population is based on the valid shared borders described above.

### Climatology of sample and study camps

Of the 17 study camps, 9 of 17 are classified by the Köppen climate classification as tropical, desert (Aw), 7 of 17 are classified as arid steppe, hot arid (BSh), and Melkadida is classified as arid desert, hot arid (BWh). The 5,000 sample border sites distributed in the same border regions as the study camps are largely similar in climatology (see Supplementary Table S1), with 75 percent of the sample sites sharing the specific three climate zones (Aw, BSh, and BWh). Of the main Köppen climate groups, more than 95 percent of the sample camps are either tropical (A) or arid (B), with the rest temperate (C).

### Exposure variable calculation and normalization

Using the Google Earth Engine API, we calculated the mean of each exposure variable within each camp boundary and the buffered sample location at a 100-m resolution. This zonal mean provides a camp-level value for all eleven variables at the 17 refugee camps and the 5,000 sampled border sites. Because of the significant differences in scale between variables, we normalized variable datasets for each refugee camp and comparison sample sites to ensure that every variable represents distinct high and low exposure values^63,64^. For each refugee camp, we use the random sample of border sites as the comparison dataset for normalization and index construction. We constructed a min–max normalization function for all variables *i* with value *x* Eq. (3) to normalize each variable.

3 $$Normalized_{i} = \frac{x - min\left( x \right)}{{max\left( x \right) - min\left( x \right) }}$$

Normalization scaled variables from 0 to 1, with the assumption that higher values of all variables (except for surface soil moisture) would contribute to a higher level of exposure^63^. For surface soil moisture (SSM), we used a flipped min–max normalization procedure Eq. (4), assuming that lower SSM would enhance exposure via a greater likelihood of desertification and lower agricultural yield.

4 $$SSM_{i} = \frac{max\left( x \right) - x}{{max\left( x \right) - min\left( x \right) }}$$

The distribution of each variable was reviewed to identify irregularities that could skew the normalization process. Nearly all variables were normally distributed around their respective national mean values with few outliers. However, the flow accumulation variable exhibited a non-normal distribution with a group of anomalously high values. These extreme values were excluded from the flow accumulation distribution through winsorization with a 99th percentile threshold^64,65^. We also identified a small amount (0.04% of observations) of missing data within study refugee camp boundaries and sampled border sites for two variables, SSM and specific humidity (Q). We addressed these missing data using mean imputation and confirmed a very slight shift in the resulting variable distribution with a very high correlation (R^2^ > 0.99) between the imputed and original dataset.

### Comparison of exposure between refugee camps and sampled border sites

We measured exposure at refugee camps and sampled border sites as the unweighted average of the eleven normalized variables Eq. (5) and calculated a simple percentile Eq. (6) and rank representing a given refugee camp's exposure relative to sampled border sites in the camp's country. We also identified the main drivers of exposure for each camp, defined as variables with one of the three highest normalized values measured at a given camp and in the top quartile of the national distribution of values at sampled border sites.

5 $$Exposure = \frac{\sum var1 + var 2 \ldots }{{11}}$$

6 $$Percentile = 1 - \frac{Exposure\;rank}{{\frac{N\;camps}{{Comparison\;border\;region}}}}*100$$

**Figure 8:**
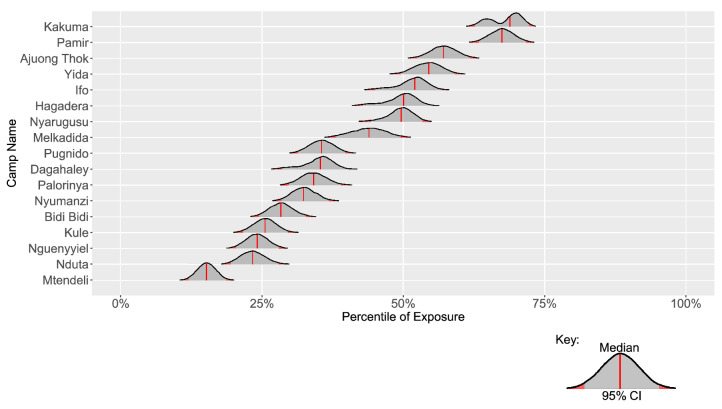
Percentile of camp exposure and confidence interval. Density plots are based on bootstrap samples of 10,000 model iterations per camp. The 95% confidence interval is shaded in gray, the observations outside of the 95% confidence interval are shaded in red, and the median exposure is indicated by a red vertical line.

To test the robustness of the exposure index, we performed a series of sensitivity analyses during and after index construction, including testing distribution differences based on the number of sampled border sites, between random samples, the spatial resolution of variable extraction, and testing different imputation strategies for missing data^64^. An additional set of indices was constructed to check for the overfitting of variables by generating exposure variants. The eleven exposure variants, *Exposure*_*A*_ through *Exposure*_*K*_, respectively represent indices based on the same eleven variable unweighted ranking scheme as above but with the exclusion of a single variable (i.e., A through K) in each overall exposure measurement (Fig. 3). The range of values for each exposure variant measured at a given refugee camp indicates the sensitivity of the exposure index for the camp and the shared border sample camps. Bootstrap samples were also generated based on 10,000 model iterations per camp (Fig. 8), with the iteration sample size based on the reference group (i.e., the number of sample border sites for each respective study camp).

## Supplementary Information

Supplementary Information.

## Data Availability

The Python code used to generate the environmental and climate data used in the exposure index, corresponding datasets, and the raw figures are available at the following GitHub repository: https://github.com/eastcoasting/Refugee-Camp-Exposure-Index.
