# Supplementary material for: Indexing climatic and environmental exposure of refugee camps with a case study in East Africa
Source: Sci Rep. 2023 May 9;13:7533. doi: 10.1038/s41598-023-31140-7 (PMC10170149; doi:10.1038/s41598-023-31140-7)
Supplement: Supplementary file 1 — Supplementary Information. [file 41598_2023_31140_MOESM1_ESM.docx]

**Indexing Climatic and Environmental Exposure of Refugee Camps with a Case Study in East Africa**

*Supplementary Information*

Michael Owen^1,^ *, Andrew Kruczkiewicz^2, 3, 4, +^, Jamon Van Den Hoek^5, +^

^1^ Columbia University, Department of Earth and Environmental Sciences, New York, 10027, USA

^2^ Columbia University, International Research Institute for Climate and Society, Climate School, New York, 10027, USA

^3^ Red Cross Red Crescent Climate Centre, The Hague, The Netherlands

^4^ University of Twente, Faculty of Geo-information Science and Earth Observation, Enschede, The Netherlands

^5^ Oregon State University, Geography and Geospatial Science Program, College of Earth, Ocean, and Atmospheric Sciences, Corvallis, 97331, USA

*m.owen@columbia.edu

+ these authors contributed equally to this work

**
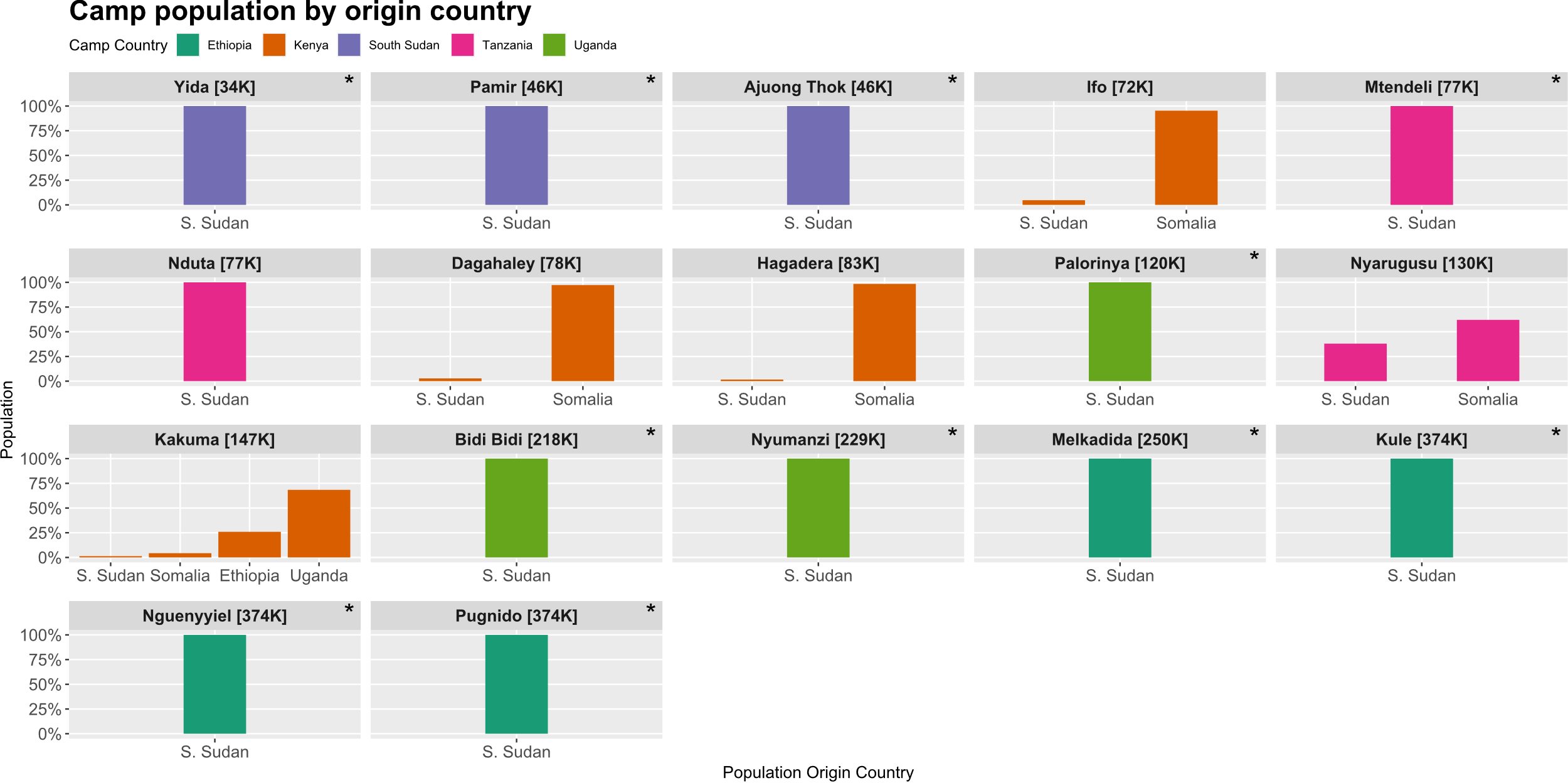
**

**Supplementary Figure 1: Camp population disaggregated by origin country.** Population sourced from the most disaggregated statistics available; where camp-level data was not available, district-level data was used (indicated by the asterisk in the facet label).

**
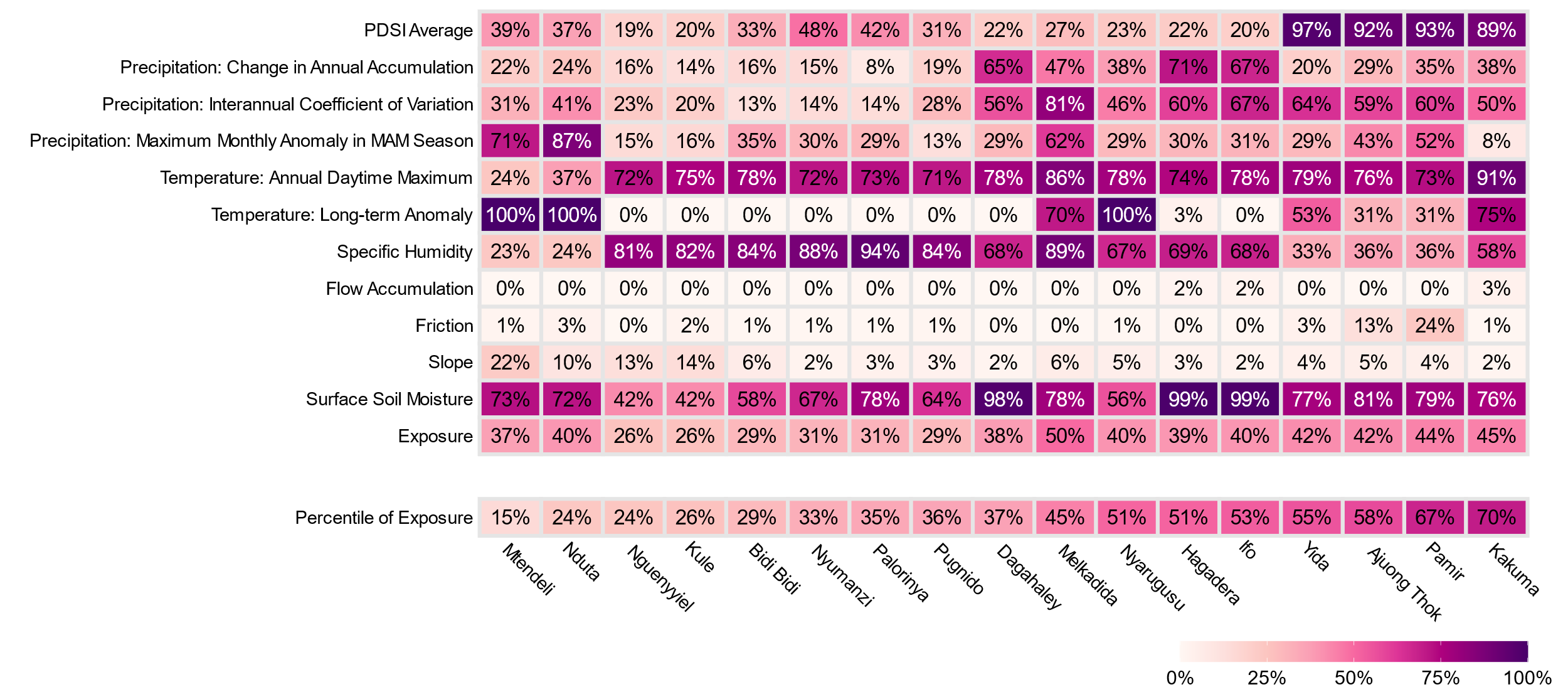
**

**Supplementary Figure 2: Underlying variable percentiles per study camp.** Heatmap of the calculated values that comprise the exposure index and percentile of exposure. Each cell represents a relative value for a given camp-variable pair, based on the comparison sample (e.g., relative to the other sample sites on the shared border of Tanzania and Burundi, Mtendeli’s PDSI Average was in the 39th percentile of all observed values).

| **Köppen Zone** | **Ethiopia** | **Kenya** | **South Sudan** | **Tanzania** | **Uganda** | **Total** |
| --- | --- | --- | --- | --- | --- | --- |
| **Tropical (A)** | **444** | **253** | **177** | **885** | **475** | **2234** |
| Desert (Aw) | 383* | 96 | 177* | 885* | 475* | 2016 |
| Fully Humid (Af) |  | 81 |  |  |  | 81 |
| Monsoonal (Am) | 61 | 35 |  |  |  | 96 |
| Steppe (As) |  | 41 |  |  |  | 41 |
|  |  |  |  |  |  |  |
| **Arid (B)** | **518** | **1672** | **323** | **3** | **22** | **2538** |
| Desert, Hot Arid (BWh) | 341* | 509 | 2 |  |  | 852 |
| Steppe, Hot Arid (BSh) | 177 | 1163* | 321* | 3 | 22 | 1686 |
|  |  |  |  |  |  |  |
| **Temperate (C)** | **38** | **75** |  | **112** | **3** | **228** |
| Desert, Hot Summer (Cwa) |  |  |  | 21 |  | 21 |
| Desert, Warm Summer (Cwb) | 3 |  |  | 91 |  | 94 |
| Fully Humid, Hot Summer (Cfa) | 3 |  |  |  |  | 3 |
| Fully Humid, Warm Summer (Cfb) | 32 | 75 |  |  | 3 | 110 |
|  |  |  |  |  |  |  |
| Total | 1000 | 2000 | 500 | 1000 | 500 | 5000 |

**Supplementary Table 1: Köppen climate classification of sample border sites.** Main climate zones—Tropical (A), Arid (B), and Temperate (C)—and totals are shown in bold. Further subclassification is based on variance in seasonal precipitation, and, for all but the tropical zone, a further classification is based on temperature/heat level. Study camp climate zones are denoted with an asterisk.
